# The influence mechanism underlying meaning in life on career adaptability among college students: a chain intermediary model

**DOI:** 10.3389/fpsyg.2024.1292996

**Published:** 2024-03-04

**Authors:** Zhengzheng Lin

**Affiliations:** College of Normal Education, Longyan University, Longyan, China

**Keywords:** meaning in life, career adaptability, positive coping styles, hope, COVID-19

## Abstract

**Introduction:**

The coronavirus disease 2019 (COVID-19) has posed a huge challenge to the career situation of college students. This study aimed to understand the mechanism underlying meaning in life on career adaptability among college students during the COVID-19 pandemic.

**Methods:**

A quantitative method was adopted. In total, 1,182 college students were surveyed using the Meaning in Life Questionnaire, the Simplified Coping Style Questionnaire, the Adult General Hope Scale, and the Career Adapt-Abilities Scale.

**Results:**

There was a significant positive correlation between meaning in life, positive coping styles, hope, and career adaptability. Positive coping styles and hope play a separate mediating role and a chain mediating role.

**Discussion:**

The findings of this study emphasize the importance of meaning in life among college students to improve their career adaptability. Furthermore, positive coping styles and increased levels of hope contribute to the development of career adaptability among college students.

## Introduction

College students' mental health deteriorated rapidly during the COVID-19 outbreak. The impact of the pandemic and related prevention and control measures, coupled with the pressures of study and employment, led to students' maladjustment, which might have a negative impact on their future careers and mental health (Conrad et al., 2021; Zhang et al., 2021). College students are at that stage in their lives where they experience the challenge of finding their “professional self.” Faced with complex and changeable employment situations, they must be well prepared. The key to career preparation for college students is improving their career adaptability, which is regarded as a core ability for career success (Hou et al., 2012). Research has shown that high levels of career adaptability can cushion the impact of environmental stressors, which in turn enhances wellbeing (Ding and Li, 2023). Therefore, it is necessary to investigate the factors influencing career adaptability to help students grow under the stress of the pandemic.

Social adjustment is produced by the interaction between the individual and the social environment, in which the individual continuously adjusts their behavior and way of thinking to the changing social environment (Dong et al., 2021). Career adaptability, on the other hand, is an expression of the competence of social adjustment in the vocational field. It is the ability to cope with career changes in their interactions with the career environment (van Vianen et al., 2009). A recent study showed that Chinese college students could not fully adapt in the face of future career changes due to the impact of COVID-19 (Wan et al., 2021). Psychological career phenomenon is a dynamic and endogenous process of change (Savickas, 2005). Intrinsic psychological factors can significantly predict career adaptability (Buyukgoze, 2016). Accordingly, this study explores the intrinsic elements that affect college students' career adaptability, providing implications for enhancing college students' career adaptability and helping them cope with uncertain employment environments so as to improve their wellbeing.

## Meaning in life, positive coping styles, and career adaptability

As Frankl (1992) states, meaning in life is a protective factor against painful events. Meaning in life is one of the most important features of wellbeing, with its core functions becoming more prominent during the pandemic (Yildirim and Arslan, 2022). Meaning in life means that the individual can clearly perceive the value of the meaning of existence, which contributes to the achievement of goals (Steger, 2012). The inability to experience meaning in life may have negative effects, such as depression and anxiety, and even extreme behaviors, such as self-injury or suicide (Sun et al., 2022). Meaning in life is relevant to student learning, life satisfaction, and career fields (To et al., 2014; Sari, 2019). The process of making decisions related to meaning in life is one of the adaptation. Maladjustment is manifested by the lack of a clear and meaningful pursuit of life, a feeling of confusion or dissatisfaction with life, and a loss of motivation to find meaning and value in one's existence. A career involves close attention and reflection on one's own survival and development, and it is in the process of career development that meaning in one's life is revealed and fulfilled.

In an epidemic, coping styles are critical to psychological wellbeing in response to the epidemic (Gurvich et al., 2020). A positive coping style is an active behavioral effort made by an individual after a cognitive judgment of changes in the internal and external environments (Zhao et al., 2017). The domain function model suggests that individuals interact with their social environment by adapting to or changing it or regulating themselves to achieve good adaptation and development. Adopting positive coping styles can help to avoid personal failure and distress, thus maintaining good social adjustment and wellbeing (Li et al., 2017; Fullerton et al., 2021).

Coping styles have been shown to mediate the relationship between personal traits (e.g., self-esteem) and career decision-making difficulties (Xu et al., 2022). Previous research suggests that college students' meaning in life has significantly and positively predicted positive coping styles; finding and experiencing meaning in life enables them to cope more positively (Aldwin, 2007). Meaning in life can support the management of negative life events (Chen et al., 2021). Individuals with higher levels of meaning in life tend to adopt positive coping styles to solve problems in response to stressful events. Empirical research has confirmed the relationship between coping styles and adaptation, which is an important predictor of an individual's physical and mental health and environmental adaptability (Barendregt et al., 2015). In students, a positive coping style partially mediates the relationship between meaning in life and the level of social adjustment. Protective coping strategies can weaken or buffer negative psychological effects (Hollister-Wagner et al., 2001; Zhao and Shi, 2016). Therefore, we proposed the following hypothesis:

H1: A positive coping style will mediate the relationship between meaning in life and career adaptability.

## Meaning in life, hope, and career adaptability

Hope theory posits that hope is a trait that has a significant impact on general wellbeing and full functioning (Snyder, 2002). Research has shown that hope can help individuals cope with environmental risk factors and overcome traumas, which is important for healthy physical and mental development (Lenz, 2020). The stress-buffering hypothesis states that positive traits moderate the potential negative effects of stress on psychological functioning and optimize event outcomes. This suggests that hope is an important mechanism mediating psychological adjustment (Cohen and Wills, 1985; Hagen et al., 2010). In this ever-changing world, hopeful individuals tend to have higher levels of career wellbeing (e.g., job satisfaction and career adaptability) to prepare for a successful life (Hirschi, 2014; Ding and Li, 2023). Yalçin and Malkoç (2015) found that meaning in life positively predicted hope among college students. College students who pursue meaning in life can increase their levels of hope. There is a significant positive correlation between meaning in life and hope, which is consistent across cultures. Consistent findings have been reported in cohort studies on Turkish adults, Malaysian nurses, and Chinese college students (Vaksalla and Hashimah, 2015; Cheng et al., 2019; Karataş et al., 2021). Individuals who can deeply experience meaning in life and strive to recognize its value have a clear perception of their lives and a sense of value in achieving their goals, thus raising their hopes. Individuals with a strong sense of meaning in life, who know the value of their existence, are better able to regulate negative emotions (Kesebir and Pyszczynski, 2014). Hope may be a mediating variable influencing psychological adjustment. Hope helps individuals achieve their desired goals, supports psychological adjustment, and positively predicts career adaptability (Buyukgoze, 2016; Deniz et al., 2023). Therefore, we proposed the following hypothesis:

H2: Hope will function as a mediator of the relationship between a sense of meaning in life and career adaptability.

## Meaning in life, positive coping styles, hope, and career adaptability

Constructing positive meaning in life can lead to effective social adjustment (Ge et al., 2021). Life that is not guided by meaning negatively impacts career tasks (Baumeister et al., 2013). By actively pursuing and gaining meaning in life, individuals focus on their future directions and show strong adaptability to their careers and lives (Yuen and Yau, 2015). Previous research has shown that positive coping styles are significantly and positively related to levels of hope. Li (2021) conducted a study on Chinese college students and found that positive coping styles contributed to higher levels of hope. Positive coping styles can facilitate access to resources, alleviate negative emotions, and enhance hope in problem solving (Chen, 2016). Therefore, we proposed the following hypothesis:

H3: Positive coping styles and hope will act as chain mediators in the relationship between a sense of meaning in life and career adaptability among college students.

**Figure 1:**
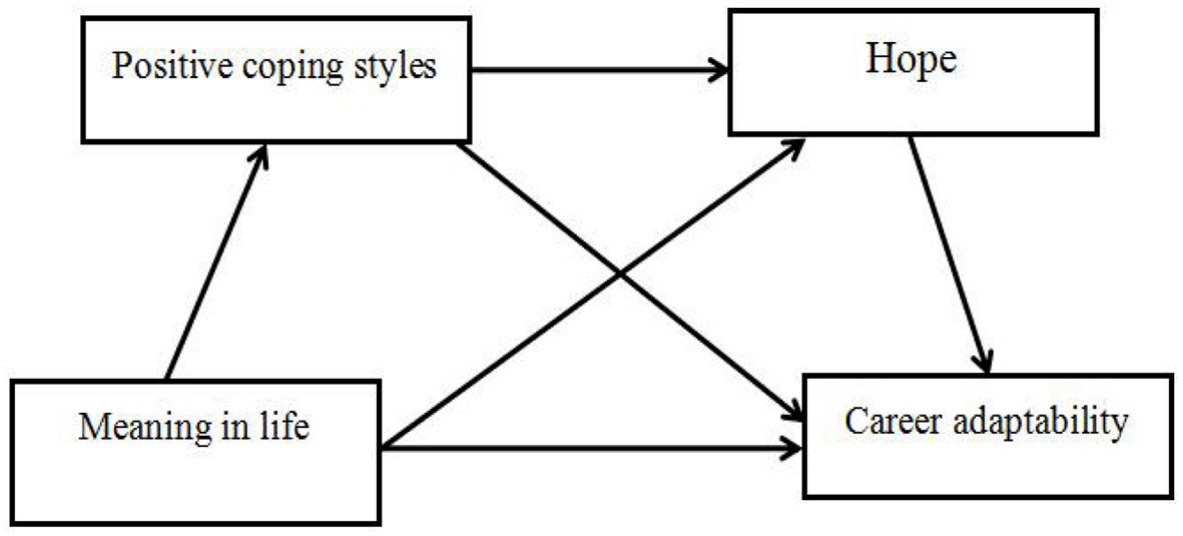
The hypothetical relationship model.

Although several studies have explored the impact of meaning in life on psychological adaptation, little research has been conducted on the mechanism underlying the role of meaning in life in career adaptability, especially in the context of COVID-19. The COVID-19 pandemic has had a negative impact on the occupational psychological health of Chinese college students. Therefore, this study enrolled college students as research participants and conducted an in-depth examination of the inner mechanism underlying the effect of meaning in life on career adaptability, contributing to the enrichment of career adaptability studies and providing suggestions for adjusting college students' career psychology during the post-pandemic period. Figure 1 provides a diagram of the hypothetical model.

## Materials and methods

### Participants

Cluster sampling was used in this study. The participants were recruited from six representative universities in Fujian, China. A total of 1,320 questionnaires were distributed; 138 questionnaires were excluded owing to missing answers, and 1,182 valid questionnaires were returned with an effective rate of 89.55%. There were 562 men and 758 women. The participants comprised 404 freshmen, 323 sophomores, 312 juniors, and 143 seniors.

### Instruments

#### Meaning in life questionnaire

This study used the Meaning in Life Questionnaire, developed by Steger et al. (2006) and revised by Liu and Gan (2010). The scale consists of nine questions (e.g., “I am looking for meaning in my life”). Items on the questionnaire are scored on a 7-point Likert scale, ranging from 1 (“strongly disagree”) to 7 (“strongly agree”). The higher the score, the higher the level of meaningfulness in life for the test. In this study, Cronbach's alpha coefficient of the scale was 0.843.

#### Adult general hope scale

We used the Adult General Hope Scale, originally developed by Snyder et al. (1996) and later translated and revised by Chen et al. (2009). The scale is divided into two dimensions, namely, the motivation factor and the path factor. There are 12 questions on the scale (e.g., “There are multiple solutions to any problem and I can think of many ways to get what is most important to me in life”). For each question, the responses are based on a 4-point Likert scale, ranging from 1 (strongly disagree) to 4 (strongly agree). Four questions regarding goals were used to divert the participant's attention and were not scored. The scale has been used extensively in China for research on hope. In the present study, Cronbach's alpha coefficient of the scale was 0.752.

#### Simplified coping style questionnaire

This study used the Positive Coping Styles subscale of the revised Simplified Coping Style Questionnaire developed by Wang and Zhang (2014), which was based on the Ways of Coping Questionnaire developed by Folkman and Lazarus (1988). The subscale has 12 questions (e.g., “when frustrated in life, I find several different ways to solve problems”) and is scored on a 4-point Likert scale. Higher scores indicate a greater tendency to adopt a positive coping style. Cronbach's alpha coefficient for the scale in this study was 0.913.

#### Career adapt-abilities scale

This study used the Career Adapt-Abilities Scale, originally developed by Savickas and Porfeli (2012) and adapted for the Chinese population by Hou et al. (2012). The Chinese version has four dimensions: career focus (“I am concerned about my career development”), career control (“I can make my own decisions”), career curiosity (“I am constantly looking for opportunities for personal growth”), and career confidence (“I will continue to solve problems through my own efforts”). Each dimension has six questions. Each response is based on a 5-point Likert scale (1 = “very unlikely,” 5 = “very likely”). Higher scores on the scale indicate higher levels of career adaptability. Cronbach's alpha coefficient of the scale in this study was 0.966.

#### Procedures

In 2022, the pandemic was effectively controlled in China, and students gradually returned to college campuses; therefore, we conducted an offline questionnaire survey. Before the commencement of the survey, the study obtained the consent of the relevant authorities. The test was administered as a group, with the questionnaire distributed uniformly, and the instructions and notes for completing the questionnaire were read by the test leader. Before completing the survey, all participants were informed of the purpose of the study and signed a voluntary informed consent form. The process was completed in ~30 min.

### Data analysis

Data were analyzed using SPSS 21.0, with the common method bias test, descriptive statistics, and correlation analysis as the main methods. Amos 21.0 was used for structural equation modeling and mediating effects testing. With meaning in life as the independent variable and career adaptability as the dependent variable, the bias-corrected percentile bootstrap method was used to test the significance of the mediating effects of positive coping style and hope, which were estimated using 95% confidence intervals. An interval estimate of 0 meant that the mediating effect was not significant, whereas the opposite implied that the mediating effect was significant.

## Results

### Common method bias test

Harman's one-way test was used to test for common bias. The results showed that eight factors had eigenvalues >1, and the variance explained by the first factor was 33.66%, which was less than the critical criterion of 40%, indicating that the common method bias of the study was relatively insignificant.

### Correlation analysis

In the Pearson correlation analysis, meaning in life, positive coping styles, hope, and career adaptability were all significantly and positively correlated (*r* = 0.44, *p* < 0.001; *r* = 0.413, *p* < 0.001; and *r* = 0.684, *p* < 0.001). Positive coping styles were significantly and positively associated with hope and career adaptability (*r* = 0.456, *p* < 0.001 and *r* = 0.417, *p* < 0.001). Hope and career adaptability were positively correlated (*r* = 0.448, *p* < 0.01). This finding indicates that when the level of meaning in life, positive coping styles, and hope increases, so does the career adaptability of college students. The results are shown in Table 1.

**Table 1:** Correlation coefficients of the variables.

	***M* ±*SD***	**MIL**	**PCS**	**Hope**	**CA**
MIL	5.14 ± 0.95	1			
PCS	2.97 ± 0.55	0.440^***^	1		
Hope	2.82 ± 0.45	0.413^***^	0.456^***^	1	
CA	3.98 ± 0.60	0.684^***^	0.417^***^	0.448^**^	1

MIL, Meaning in life; PCS, Positive coping styles; CA, Career adaptability, ^**^p < 0.01, ^***^p < 0.001.

### Measurement model

The measurement model included 4 latent variables (meaning in life, positive coping styles, hope, and career adaptability) and 11 observed variables (latent variable dimensions). Because positive coping styles are unidimensional, to reduce inflation error, a structural factorial algorithm was used to pack positive coping styles into three dimensions. First, a factor analysis was conducted, and the items were ranked from the highest to the lowest loadings and then rotated from the highest to the lowest according to the number of groups. The items with the second-highest loadings were then added sequentially in the opposite direction for balancing (Rogers and Schmitt, 2004). When the sample size is large, the χ^2^/df criterion may increase. According to Hou et al. (2004) suggestion, the χ^2^/df criterion is not recommended when the sample size is *N* ≧ 1000. The measurement model achieved a good fit (RMSEA = 0.065, GFI = 0.965, RMR = 0.014, AGFI = 0.94, NFI = 0.974, and CFI = 0.978). The intermediary model can be further analyzed.

### Intermediary model analysis

As shown in Table 2, in the results of the regression analysis, controlling for gender and grade, meaning in life positively and significantly predicted career adaptability (β = 0.438, *p* < 0.001), positive coping style (β = 0.255, *p* < 0.001), and predicted hope (β = 0.125, *p* < 0.001) and positive coping style positively and significantly predicted hope (β = 0.281, *p* < 0.001). When gender, grade, meaning in life, positive coping style, and hope were entered into the regression equation simultaneously, meaning in life, positive coping style, and hope significantly predicted career adaptability.

**Table 2:** Regression analysis between variables.

**Dependent variables**	**Independent variables**	***R***	***R^2^***	***F***	**β**	***t***
CA	Gender	0.684	0.468	345.089^***^	−0.009	−0.34
	Grade				0.007	0.564
	MIL				0.438	32.174^***^
PCS	Gender	0.442	0.195	95.124^***^	−0.002	−0.064
	Grade				−0.021	−1.501
	MIL				0.255	16.785^***^
Hope	Gender	0.526	0.277	112.684^***^	−0.101	−4.554^***^
	Grade				−0.004	−0.369
	MIL				0.125	9.602^***^
	PCS				0.281	12.314^***^
CA	Gender	0.712	0.506	241.385^***^	0.015	0.612
	Grade				0.011	0.94
	MIL				0.368	24.252^***^
	PCS				0.094	3.535^***^
	Hope				0.231	7.214^***^

MIF, Meaning in life; PCS, Positive coping style; CA, Career adaptability, ^***^ p < 0.001.

**Figure 2:**
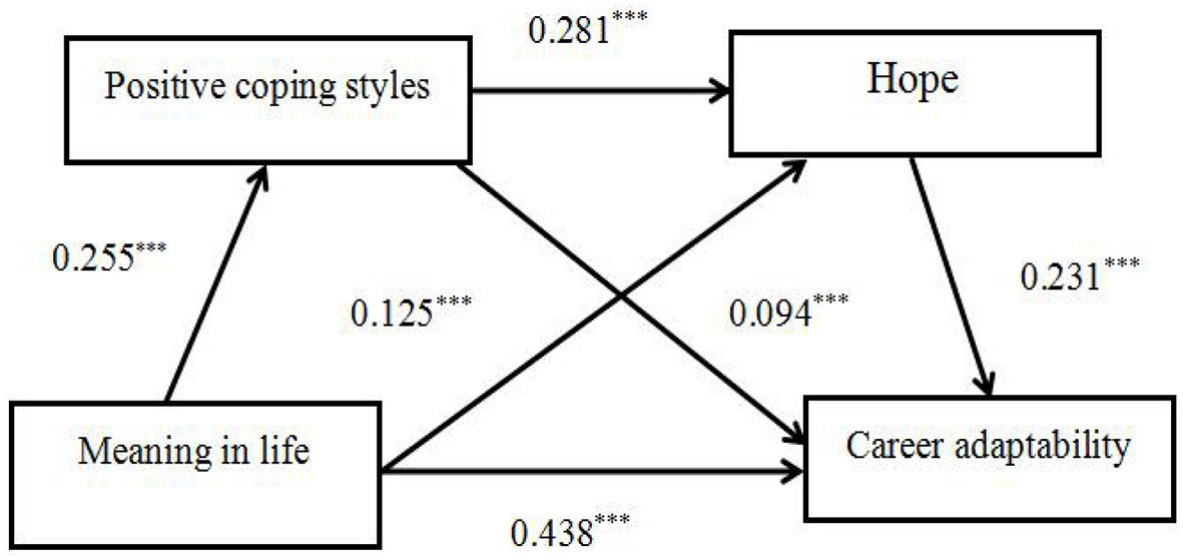
Diagram of chain mediation. ***p < 0.001.

The bias-corrected percentile bootstrap method was further used to test the mediating effects, and the results showed that the indirect effects of each path in the model were significant. The results are summarized in Table 3. The mediating effect of positive coping styles and hope was significant, with a total indirect effect value of 0.07. The mediating effect was generated by three chains of mediators. Indirect Effect 1 consisted of meaning in life, positive coping styles, and career adaptability; the indirect effect was 0.024, and the bootstrap 95% confidence interval did not contain 0 (CI = [0.013, 0.065]), indicating that the mediating effect of positive coping styles was significant. Indirect Effect 2 consisted of meaning in life, hope, and career adaptability; the indirect effect was 0.029, and the bootstrap 95% confidence interval did not contain 0 (CI = [0.025, 0.069]), suggesting a significant mediation effect of hope. Finally, Indirect Effect 3 consisted of meaning in life, positive coping styles, hope, and career adaptability, with an indirect effect of 0.017, showing significant mediating effects. The pathways through which college students' meaning in life affected their career adaptability are shown in Figure 2.

**Table 3:** Mediating effects of positive coping style and hope on the relationship between meaning in life and career adaptability.

	**Effect**	***Boot SE***	***BootLLCI***	***BootULCI***
MIL → PCS → CA	0.024	0.013	0.013	0.065
MIL → Hope → CA	0.029	0.011	0.025	0.069
MIL → PCS → Hope → CA	0.017	0.006	0.015	0.038

MIL, Meaning in life; PCS, Positive coping styles; CA, Career adaptability.

## Discussion

This study explored the mediating role of positive coping styles and hope in the relationship between college students' meaning in life and career adaptability. The study findings suggest that meaning in life, positive coping styles, and hope can positively influence college students' career adaptation even during the COVID-19 epidemic phase.

First, the influence of meaning in life on career adaptability was realized through the influence of positive coping styles, which was similar to the findings of Failo et al. (2018) and confirmed H1. The COVID-19 pandemic triggered a global public health crisis, and people experienced difficult times. Meaning in life plays an important role in coping with stress in difficult situations (Rosenberg, 2020), such as the pandemic. The domain function model suggests that individuals interact with their social environment by adapting to or changing it or regulating themselves to achieve good adaptation and development. By adopting multiple coping strategies, individuals eventually reach equilibrium with their environment (Zou et al., 2012; Li et al., 2017). Positive coping strategies (such as talking, seeking advice from others, and changing negative perceptions) help individuals improve their ability to deal with problems and create a virtuous cycle (Goodvin and Romdall, 2013). Individuals with a high level of meaning in life are more likely to choose positive ways of coping with life challenges, have positive perceptions in the face of difficulties and setbacks, adopt effective coping strategies, and adapt to changes in the external environment by solving problems or seeking help from the outside, thus enabling them to adapt better (Hajitabar Firouzjaee et al., 2019; Ward et al., 2022).

Our study's findings on the mediating role of hope in meaning in life and career adaptability are consistent with previous research (Korkmaz and Önder, 2019) and confirmed H2. Hope is an important psychological resource for career development and is associated with positive workplace outcomes. Despite the threat of recurring outbreaks, hope remains an important positive trait in reducing negative psychological impacts (Yildirim and Güler, 2021). Individuals with a high level of meaning in life can correctly evaluate their own abilities and values, have a high sense of self-worth, pursue their own life and beliefs about it, and strive for a sense of value in the future, enhancing their cognition and belief in moving toward their goals and experiencing self-improvement and self-fulfillment, thus increasing hope. Individuals with high levels of hope also have a desire for a career, have a positive self-commitment to accept, and integrate changes in the environment more readily, which, in turn, inspires them to be more adaptable to their careers (Wandeler and Bundick, 2011; Hirschi, 2014).

Finally, positive coping styles and hope acted as chain mediators in the relationship between meaning in life and career adaptability, which confirmed H3. This finding suggests that positive coping styles and hope are potential mechanisms underlying the relationship between meaning in life and career adaptability. The stress-buffering model indicated that protective factors (e.g., meaning in life and hope) can diminish the negative impact of risk factors (e.g., epidemics). The level of meaning in life is an important factor for psychological wellbeing, especially during crises (Arslan et al., 2022). If college students actively seek and experience meaning in their lives, they are more likely to adopt positive coping strategies and use effective methods to overcome unfavorable situations, prompting them to pursue their goals, believing in their ability to achieve them, and reaping higher levels of hope when putting into practice. Hope is also considered a protective factor for psychological adaptation during COVID-19 (Seher and Zeynep, 2023). The high levels of hope help individuals prepare for future challenges, have a clear plan for their careers, adapt to career changes in an organized manner, and quickly integrate into a changing career environment, which is also a concrete expression of greater career adaptability. A high level of meaning in life promotes an optimistic attitude toward life and positive behavioral coping patterns to effectively adapt to one's future career. Higher hope indicates better adjustment, adaptability, and strong competence in solving career problems. This suggests that positive coping styles and hope are effective paths for enhancing career adaptability.

## Practical implications

Enhancing career adaptability is important to protect the wellbeing of college students. Higher education institutions can adopt targeted strategies to support students' career development during future pandemics.

First, students should be guided to deepen their understanding of the value of life through their experiences of fighting the COVID-19 pandemic to face the unpredictability of a career environment with an optimistic attitude toward life. Students need to be guided in their perception and exploration of meaning in life and to awaken to a sense of responsibility for their future. They should be encouraged to actively seek direction and value in life, closely integrate the meaning of existence with their career, pay attention to their own career development, establish career goals, and keep abreast of all career information.

Additionally, the pandemic has been both a crisis and an opportunity. Coping styles become crucial in the face of career uncertainty (Gurvich et al., 2020). A proactive coping style can help students effectively address career adjustment dilemmas. Students should be encouraged to accept the uncertainty in their careers, develop their ability to identify and seize opportunities, and think of strategies to respond to “opportunity events” to develop their careers. For example, schools can create platforms for students to engage in more social practice activities, where they can deepen their understanding of themselves and their external environment, accumulate career experience, and improve their ability to regulate their careers.

Finally, goals are the central element of hope (Snyder, 2002). Career goals that are too high and beyond students' ability to achieve can leave them frustrated. If goals are too low and easy to achieve, it will reduce students' sense of value and meaning. Therefore, students should be guided to form reasonable expectations regarding their careers and refine their goals.

## Limitations and future prospects

This study has several limitations. First, most senior-year students were enrolled in off-campus internships and not at school. This resulted in a small sample size of senior-year students when the questionnaire was collected. This may affect the applicability of the results. In the future, a senior-year student group should be selected as a sample to verify the reliability of the results and expand the generalizability of the findings. Second, there may be other factors influencing career adaptability, such as employment pressure and social support, and these factors can be explored in depth in future studies. Additionally, we used a cross-sectional study to examine the factors affecting career adaptability; however, we cannot judge the causality between the variables. Future longitudinal follow-up studies should be conducted to better understand the relationship between meaning in life, positive coping styles, hope, and career adaptability. It is also important to compare the results during the pandemic with the end of the epidemic to explore the stability of the relationship between the four variables. Finally, the research method of the questionnaire survey may have been influenced by bias. There may be problems in answering truthfully, which may have affected the validity of the research. Future studies should adopt both experimental and qualitative methodologies to expand and improve on this area of research.

## Conclusion

The impact of the coronavirus pandemic on people's studies and work will probably continue for some time. In this study, we found that positive coping style and hope act as a chain mediator between meaning in life and career adaptability. These findings show that meaning in life, positive coping style, and hope are important characteristics that enhance career adaptability. Furthermore, the findings have implications for career education for college students in the post-epidemic era or any future pandemic.

## Data availability statement

The raw data supporting the conclusions of this article will be made available by the authors, without undue reservation.

## Ethics statement

The studies involving humans were approved by the Ethics Committee of Longyan University. The studies were conducted in accordance with the local legislation and institutional requirements. The participants provided their written informed consent to participate in this study.

## Author contributions

ZL: Writing – original draft, Writing – review & editing.
